# Radiographic bone resorption after reverse total shoulder arthroplasty using a single onlay platform: risk factors and distribution patterns in a Japanese cohort

**DOI:** 10.1016/j.jsea.2026.100053

**Published:** 2026-06-25

**Authors:** Taisuke Nozu, Yoshihiro Hirakawa, Tomoya Manaka, Katsumasa Nakazawa, Yoichi Ito, Hisayoshi Kato, Yasuhiro Nakane, Hidetomi Terai

**Affiliations:** a Department of Orthopaedic Surgery, Osaka Metropolitan University, Graduate School of Medicine, Osaka, Japan; b Ishikiriseiki Hospital, Higashi-Osaka, Japan; c Ito Clinic, Osaka Shoulder Center, Matsubara, Japan; d Department of Orthopedic Surgery, Nippon Kokan Fukuyama Hospital, Fukuyama, Japan; e Sumiya Orthopaedics Hospital, Wakayama, Japan

**Keywords:** Reverse total shoulder arthroplasty, Humeral stem, Short stem, Standard stem, Bone resorption, Stress shielding, Canal filling ratio, Propensity score matching

## Abstract

**Background:**

Bone resorption around the humeral stem is a frequent radiographic finding after reverse total shoulder arthroplasty (rTSA). Short stems have been introduced to reduce stress shielding and preserve proximal bone, but comparative data in Asian populations are limited. We evaluated the incidence, distribution, and factors associated with humeral bone resorption among Japanese patients who underwent cementless rTSA using a single onlay humeral platform and compared standard and short stems.

**Methods:**

This multicenter retrospective study included shoulders treated with cementless rTSA in Japan. After excluding 6 shoulders with incomplete data, 128 shoulders were eligible. Propensity score matching was performed based on age, sex, body mass index, diagnosis, and pre-operative range of motion. Bone resorption in the lateral metaphysis, lateral diaphysis (LD), medial metaphysis, and medial diaphysis zones was graded using the Inoue classification. Canal filling ratios were calculated at metaphyseal and diaphyseal levels, and stem alignment was categorized as valgus, neutral, or varus. Multivariable logistic regression identified factors associated with grade ≥3 bone resorption.

**Results:**

After propensity score matching, baseline characteristics were balanced between the standard stem (n = 35) and short stem (n = 35) groups. At 2 years post-operatively, grade ≥3 bone resorption was observed more often with standard stems (23 of 35 shoulders, 65.7%) than with short stems (13 of 35 shoulders, 37.1%; unadjusted *P* = .03). The LD zone most frequently exhibited resorption and differed significantly between groups after correction for multiple secondary comparisons (standard: 12 of 35 shoulders, 34.3%; short: 2 of 35 shoulders, 5.7%; unadjusted *P* < .01), whereas the lateral metaphysis, medial metaphysis, and medial diaphysis zones did not differ significantly. Compared with the nonresorption group, the resorption group included more female patients (52.8% vs. 20.6%; unadjusted *P* < .01). Age, body mass index, canal filling ratios, and alignment did not differ between these groups. Female sex (odds ratio, 3.64; 95% confidence interval, 1.07–12.37; *P* = .04) and standard stem use (odds ratio, 5.54; 95% confidence interval, 1.19–25.87; *P* = .03) were independently associated with resorption. Range of motion at 2 years did not differ according to resorption status.

**Conclusion:**

In this Japanese cohort treated with a single onlay humeral platform, grade ≥3 bone resorption occurred more frequently with standard stems than with short stems, particularly in the LD zone. Female sex and standard stem use were independently associated with resorption.

Reverse total shoulder arthroplasty (rTSA) has become a reliable treatment for cuff tear arthropathy and irreparable rotator cuff tears, providing consistent pain relief and functional improvement.^2^^,^^31^ Despite these clinical gains, radiographic remodeling around the humeral component has been reported.^3^^,^^8^^,^^10^^,^^21^^,^^26^ Such remodeling is commonly interpreted as stress shielding-related bone resorption and is frequently asymptomatic during the early post-operative course^2^^,^^21^; however, it may compromise proximal bone stock for revision, influence fixation durability, and potentially contribute to periprosthetic events in the long term.^11^^,^^12^^,^^25^ In the literature, the incidence and anatomic distribution of remodeling exhibit considerable heterogeneity, particularly within the proximal metaphysis. This variability is likely attributable to differences in implant geometry, coating distribution, fixation philosophy, canal occupancy, radiographic definitions, and length of follow-up.^8^^,^^9^^,^^15^^,^^22^^,^^26^

Previous studies have identified both patient-related and implant-related factors associated with bone resorption. Patient-related factors such as female sex and osteoporosis have been repeatedly implicated; implant-related factors include a higher canal filling ratio (FR), longer humeral stems, cementless fixation, onlay humeral designs, and an increased neck-shaft angle.^5^^,^^6^^,^^8^^,^^9^^,^^15^^,^^16^^,^^22^^,^^26^^,^^33^ Although studies focused on alignment are limited, several have suggested that coronal malalignment may be associated with bone resorption, possibly by promoting cortical contact and altering load transfer.^4^^,^^27^ Furthermore, short stems, which were developed to preserve proximal bone via metaphyseal fixation, may be more susceptible to coronal malalignment than standard stems.^1^^,^^9^ Recent studies have also demonstrated substantial heterogeneity in the anatomic distribution of stress shielding across implant systems, supporting the concept that this phenomenon may be strongly implant dependent.^1^^,^^9^^,^^29^ Therefore, evaluation using a single implant platform is important to reduce design-related heterogeneity when assessing the influence of stem length, alignment, and canal filling.

Asian patients may differ from Western patients in humeral canal morphology, cortical thickness, body size, and bone quality, which may influence stem filling behavior, fixation mechanics, and stress shielding patterns after rTSA.^15^^,^^32^ However, Asian populations remain under-represented in studies evaluating humeral bone remodeling after rTSA. Therefore, we conducted a multicenter investigation of a Japanese cohort using a single onlay platform (Exactech Equinoxe standard stem or press-fit short stem) after propensity score matching to reduce measured confounding. Using this uniform system, we aimed to evaluate the 2-year post-operative incidence and anatomic distribution of bone resorption and identify factors associated with grade ≥3 bone resorption. We hypothesized that stem design and coronal alignment characteristics may influence the pattern and incidence of zone-specific bone resorption after rTSA.

## Materials and methods

### Patient cohort

We used data from cases registered in the Shoulder Association of Multiunit with Rotator Cuff and Arthritis Investigation database, a multicenter collaborative shoulder disorder database constructed using Research Electronic Data Capture (REDCap; Vanderbilt University, Nashville, TN, USA; http://project-redcap.org). Patients who underwent primary rTSA between April 2015 and March 2022 for irreparable massive rotator cuff tears or cuff tear arthropathy were included. Patients with primary glenohumeral osteoarthritis or inflammatory arthritis were not included because the present study focused on rTSA performed for rotator cuff-related pathology. The exclusion criteria were revision arthroplasty, fractures, rheumatoid arthritis, infection, previous surgery, additional tendon transfers, and cemented fixation. Institutional review board approval was obtained from each institution, and all patients provided written informed consent. The reporting of this study followed the Strengthening the Reporting of Observational Studies in Epidemiology (STROBE) recommendations for observational studies.

### Implant description

All humeral components used in this study were cementless components from a single onlay humeral platform (Exactech Equinoxe system; Exactech Inc., Gainesville, FL, USA). Patients received either the standard stem (Exactech Equinoxe; Exactech Inc.) or the press-fit short stem (Equinoxe Preserve humeral stem; Exactech Inc.). The standard stem was available in lengths of 110–125 mm in 2.5-mm increments, and was designed to achieve metaphyseal and diaphyseal fixation. The short stem had a fixed length of 70 mm and a metaphyseal-engaging press-fit design with reduced distal canal occupation. Both stems had wedge-shaped geometry, proximal porous coating for bone ongrowth, and a tapered distal portion. All humeral stems had a neck-shaft angle of 145°. Stem size was determined intraoperatively by sequential broaching to achieve rotational stability and appropriate cortical contact without excessive canal overfilling. Baseplate configuration, glenosphere size (36, 38, or 40 mm), and glenoid-side lateralization were selected according to patient anatomy, glenoid bone morphology, and soft tissue tension at the discretion of the operating surgeon.

### Surgical technique

All procedures were performed by fellowship-trained shoulder surgeons at 4 centers. General anesthesia was administered, and the procedure was performed with the patient in the beach-chair position. All patients received interscalene blocks for post-operative pain control, and a deltopectoral surgical approach was used. The cephalic vein was mobilized laterally. The subscapularis was managed with tenotomy, and repair was performed when tendon quality and intraoperative mobility allowed stable repair without excessive tension. The humerus was osteotomized in 20° of retroversion. A glenoid baseplate was implanted at the inferior edge of the glenoid surface. During post-operative rehabilitation, an abduction sling or shoulder immobilizer was worn for 2 weeks after surgery. Passive motion exercises were initiated early during the post-operative period. Active range of motion (ROM) exercises were then initiated according to the patient's pain tolerance.

### Radiographic assessment

**Figure 1:**
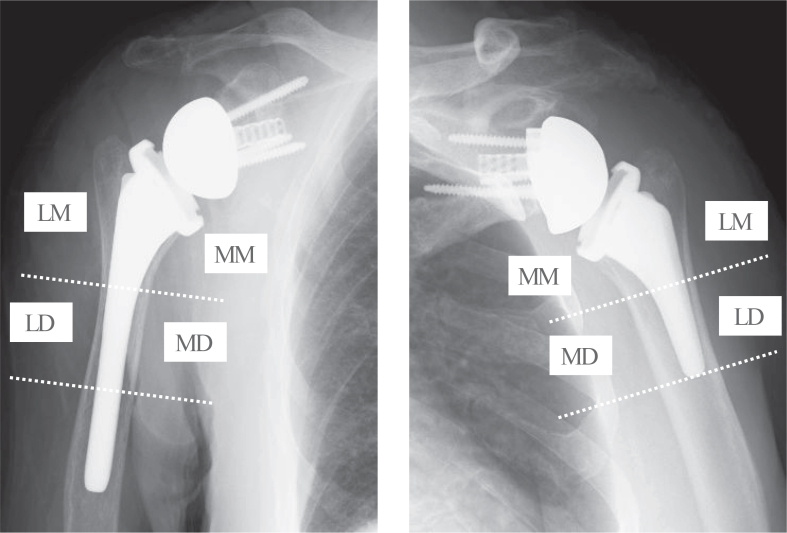
Bone resorption site. *LM*, lateral metaphysis; *LD*, lateral diaphysis; *MM*, medial metaphysis; *MD*, medial diaphysis.

**Figure 2:**
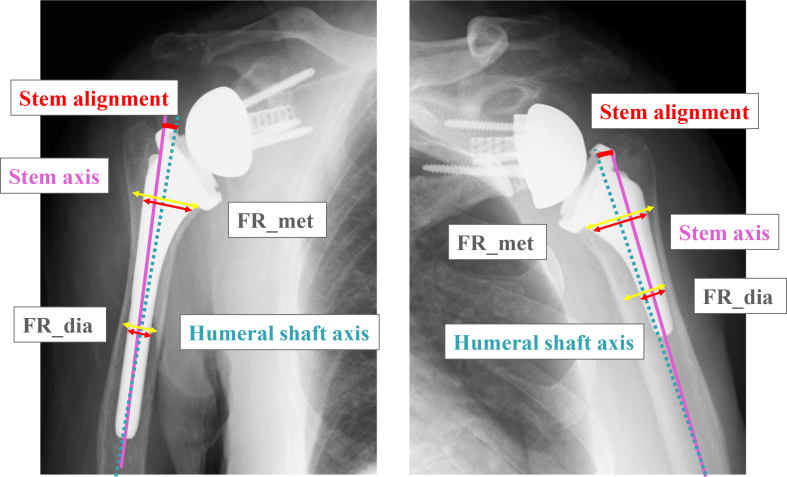
Radiographic parameters. The canal filling ratio was calculated as stem width divided by endosteal canal width at the metaphyseal (FR_met) and diaphyseal (FR_dia) levels. Coronal alignment was measured as the angle between the stem axis and humeral shaft axis.

Standardized anteroposterior views were obtained immediately post-operatively and 2 years post-operatively. The presence of proximal humeral bone resorption was determined by comparing images obtained at these time points. The proximal humerus was divided into the medial metaphysis (MM), lateral metaphysis (LM), medial diaphysis (MD), and lateral diaphysis (LD) zones (Fig. 1).^21^^,^^26^ Bone resorption severity was graded using the Inoue classification.^15^ Based on the immediate post-operative anteroposterior view, the canal FR was measured at the metaphyseal (FR_met) and diaphyseal (FR_dia) levels. Using a line perpendicular to the humeral shaft or stem axis at standardized landmarks (metaphyseal level near the humeral tray and diaphyseal level near the distal third of the stem) and following established methods, the FR at each level was calculated as stem width divided by endosteal canal width (Fig. 2).^1^^,^^23^ Coronal alignment was measured on the same image as the angle between the stem axis and humeral shaft axis and was categorized as neutral (within ±5°), valgus (>+5°), or varus (<−5°) (Fig. 2).^1^^,^^23^ All abbreviations and measurement definitions are provided in the corresponding figure legends.

### Clinical evaluation

Patient demographic data, including age at the time of surgery, sex, height, body weight, and diagnosis, were obtained from the Shoulder Association of Multiunit with Rotator Cuff and Arthritis Investigation database. Clinical data on active forward flexion, abduction, external rotation at the side, and internal rotation were collected pre-operatively and 2 years post-operatively. Internal rotation was measured by assessing the extent to which the patient could reach behind the back with the thumb and was evaluated using a 6-point scale to obtain the Constant internal rotation score. Active ROM was evaluated using a goniometer.

### Statistical analysis

All analyses were performed in R (R Foundation for Statistical Computing, Vienna, Austria). Categorical variables were compared using the chi-square or Fisher exact test. Continuous variables were compared using the *t* test or Mann-Whitney *U* test. In the propensity-matched cohort, paired comparisons were conducted using the McNemar test for dichotomous variables and the Wilcoxon signed-rank test for continuous variables. To identify factors associated with grade ≥3 bone resorption, we performed multivariable logistic regression and reported odds ratios (ORs) and 95% confidence intervals (CIs). Given the limited sample size, the number of variables entered into the multivariable model was restricted to clinically relevant variables and variables with potential biomechanical relevance. Because multiple secondary comparisons were performed, the Benjamini-Hochberg false discovery rate correction was applied to the secondary univariate and radiographic comparisons. The multivariable model was treated as the primary inferential analysis and was not adjusted for multiplicity. The findings were interpreted as associative rather than causal. A *P* value < .05 was considered statistically significant.

## Results

### Patients

**Figure 3:**
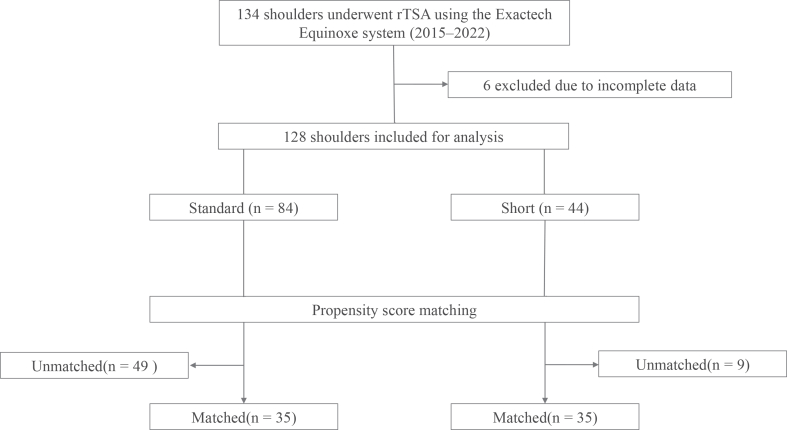
Propensity score matching.

Of the 134 patients who underwent rTSA, 6 were excluded because of incomplete data; therefore, 128 patients were included in the final analysis. Propensity score matching based on age, sex, body mass index, diagnosis, pre-operative forward flexion, pre-operative abduction, pre-operative external rotation, and pre-operative internal rotation resulted in a total of 70 shoulders (standard stem, n = 35; short stem, n = 35) (Fig. 3). No significant between-group differences in matched baseline characteristics were observed (Table I).

**Table I:** Baseline characteristics after propensity score matching.

Variable	Standard stem (n = 35)	Short stem (n = 35)	*P* value
Age (yr)	76.7 ± 5.7	75.8 ± 8.3	.58
Sex (male/female)	22 (62.9%)/13 (37.1%)	22 (62.9%)/13 (37.1%)	1.00
BMI (kg/m^2^)	23.2 ± 3.8	23.2 ± 3.2	.98
Diagnosis (CTA/RCT)	29 (82.9%)/6 (17.1%)	29 (82.9%)/6 (17.1%)	1.00
Pre-operative forward flexion (°)	77 ± 41	73 ± 39	.68
Pre-operative abduction (°)	69 ± 38	68 ± 33	.86
Pre-operative external rotation (°)	24 ± 22	19 ± 18	.24
Pre-operative internal rotation (Constant score)	4.4 ± 2.9	3.9 ± 2.5	.41

*BMI*, body mass index; *CTA*, cuff tear arthropathy; *RCT*, rotator cuff tear.

Values are presented as mean ± standard deviation or number (percentage).

In the propensity-matched cohort, paired comparisons were performed using the McNemar test for dichotomous variables and the Wilcoxon signed-rank test for continuous variables, as appropriate.

*P* < .05 was considered statistically significant.

### Incidence and distribution of bone resorption

At 2 years post-operatively, grade ≥3 bone resorption was observed in 23 of 35 shoulders (65.7%) with standard stems and 13 of 35 shoulders (37.1%) with short stems (unadjusted *P* = .03). The LD zone was the most commonly involved zone and differed significantly between the standard and short stem groups after Benjamini-Hochberg correction for secondary comparisons (standard: 12 of 35 shoulders, 34.3%; short: 2 of 35 shoulders, 5.7%; unadjusted *P* < .01). The LM, MM, and MD zones did not differ significantly between groups (Table II). Accordingly, the site-specific LD finding was emphasized as the most robust radiographic difference, whereas the overall grade ≥3 comparison was interpreted as exploratory.

**Table II:** Incidence and site of bone resorption and radiographic parameters.

Variable	Standard stem (n = 35)	Short stem (n = 35)	*P* value
Bone resorption ≥ grade 3 (n, %)	23 (65.7%)	13 (37.1%)	.03∗
LM resorption (n, %)	10 (28.6%)	10 (28.6%)	1.00
LD resorption (n, %)	12 (34.3%)	2 (5.7%)	<.01∗†
MM resorption (n, %)	11 (31.4%)	4 (11.4%)	.08
MD resorption (n, %)	1 (2.9%)	0 (0.0%)	1.00
FR_met (mean ± SD)	0.68 ± 0.08	0.73 ± 0.09	.02∗
FR_dia (mean ± SD)	0.71 ± 0.09	0.60 ± 0.10	<.01∗†
Alignment (°; mean ± SD)	1.14 ± 1.29	2.30 ± 3.30	.06
Alignment category (n, %)	Valgus 1 (2.9%), neutral 34 (97.1%), varus 0 (0.0%)	Valgus 10 (28.6%), neutral 24 (68.6%), varus 1 (2.9%)	<.01∗†

*LM*, lateral metaphysis; *LD*, lateral diaphysis; *MM*, medial metaphysis; *MD*, medial diaphysis; *SD*, standard deviation.

Bone resorption was evaluated according to the Inoue classification; grade ≥3 was defined as significant resorption.

FR_met and FR_dia represent the canal filling ratios at the metaphyseal and diaphyseal levels.

Alignment was measured relative to the humeral shaft axis; valgus and varus were defined as > +5° and < −5°, respectively, and neutral as −5° to +5°.

Unadjusted *P* values are shown. Secondary radiographic comparisons were interpreted using the Benjamini-Hochberg false discovery rate procedure.

∗ Unadjusted *P* < .05.

† Significant after Benjamini-Hochberg correction. The overall grade ≥3 comparison was interpreted as exploratory.

### Canal filling and alignment

The FR_met was lower with standard stems than with short stems (0.68 ± 0.08 vs. 0.73 ± 0.09; unadjusted *P* = .02). The FR_dia was higher with standard stems than with short stems and remained significant after Benjamini-Hochberg correction (0.71 ± 0.09 vs. 0.60 ± 0.10; unadjusted *P* < .01). The mean alignment angle did not differ significantly between groups (standard: 1.14° ± 1.29°; short: 2.30° ± 3.30°; *P* = .06); however, the alignment category distribution differed significantly and remained significant after correction (standard: valgus, 1 of 35 shoulders, 2.9%; neutral, 34 of 35 shoulders, 97.1%; varus, 0 of 35 shoulders, 0%; short: valgus, 10 of 35 shoulders, 28.6%; neutral, 24 of 35 shoulders, 68.6%; varus, 1 of 35 shoulders, 2.9%; unadjusted *P* < .01) (Table II). The FR_met comparison was therefore interpreted descriptively.

### Comparison based on the bone resorption grade

Compared with the nonresorption group, the resorption group included more female patients, and this difference remained significant after Benjamini-Hochberg correction (52.8% vs. 20.6%; unadjusted *P* < .01). Age, body mass index, FR_met, FR_dia, alignment angle, and alignment category did not differ significantly between the resorption and nonresorption groups (Table III). Given the number of secondary comparisons, nonsignificant findings should be interpreted cautiously.

**Table III:** Comparison between cases with and without bone resorption (≥ grade 3).

Variable	Bone resorption ≥ grade 3 (n = 36)	No resorption (n = 34)	*P* value
Age (yr)	76.2 ± 5.3	76.3 ± 8.7	.95
Sex (male/female, n, %)	Male 17 (47.2%), female 19 (52.8%)	Male 27 (79.4%), female 7 (20.6%)	<.01∗†
BMI (kg/m^2^)	23.5 ± 3.9	22.9 ± 3.1	.43
Stem (n, %)	Standard 23 (63.9%), short 13 (36.1%)	Standard 12 (35.3%), short 22 (64.7%)	.03∗
FR_met (mean ± SD)	0.72 ± 0.09	0.69 ± 0.09	.14
FR_dia (mean ± SD)	0.67 ± 0.10	0.63 ± 0.11	.08
Alignment (°; mean ± SD)	1.9 ± 2.0	1.6 ± 3.0	.66
Alignment (n, %)	Valgus 6 (16.7%), neutral 30 (83.3%), varus 0 (0.0%)	Valgus 5 (14.7%), neutral 28 (82.4%), varus 1 (2.9%)	.58

*BMI*, body mass index; *SD*, standard deviation.

Alignment was measured relative to the humeral shaft axis; valgus and varus were defined as > +5° and < −5°, respectively, and neutral as −5° to +5°.

Categorical variables were compared using the chi-square or Fisher exact test. Continuous variables were compared using the *t* test or Mann-Whitney *U* test, as appropriate.

Unadjusted *P* values are shown. Secondary univariate comparisons were interpreted using the Benjamini-Hochberg false discovery rate procedure.

∗ Unadjusted *P* < .05.

† Significant after Benjamini-Hochberg correction.

### Multivariable analysis

Female sex (OR, 3.64; 95% CI, 1.07–12.37; *P* = .04) and standard stem use (OR, 5.54; 95% CI, 1.19–25.87; *P* = .03) were independently associated with grade ≥3 resorption; however, FR_met and FR_dia were not significantly associated with resorption (Table IV).

**Table IV:** Multivariable logistic regression analysis for factors associated with bone resorption (≥ grade 3).

Variable	Odds ratio	95% CI lower	95% CI upper	*P* value
Sex (female)	3.64	1.07	12.37	.04∗
FR_met	1.7	0.75	3.82	.20
FR_dia	0.97	0.51	1.84	.93
Stem type (standard)	5.54	1.19	25.87	.03∗

*CI*, confidence interval.

FR_met and FR_dia represent the canal filling ratios at the metaphyseal and diaphyseal levels.

∗ *P* < .05 was considered statistically significant.

### Two-year range of motion

Forward flexion, abduction, external rotation, and internal rotation at 2 years post-operatively did not differ significantly between shoulders with and without bone resorption (Table V).

**Table V:** Comparison of post-operative range of motion (2 years) between cases with and without bone resorption (≥ grade 3).

Variable	Bone resorption ≥ grade 3 (n = 36)	No resorption (n = 34)	*P* value
Forward flexion (°)	126 ± 19	122 ± 23	.44
Abduction (°)	116 ± 25	117 ± 31	.93
External rotation (°)	32 ± 13	32 ± 17	.93
Internal rotation (Constant score)	6.6 ± 2.6	5.4 ± 3.1	.08

*P* < .05 was considered statistically significant.

## Discussion

This multicenter, propensity-matched study involving a single onlay humeral platform demonstrated that grade ≥3 humeral bone resorption at 2 years post-operatively occurred more frequently with standard stems than with short stems. The remodeling distribution also differed according to stem design: LD involvement was significantly more frequent with standard stems, whereas LM involvement occurred at a similar rate with standard and short stems. In the multivariable analysis, female sex and standard stem use were independently associated with grade ≥3 resorption; however, canal FRs and coronal malalignment were not. These findings refine our initial hypothesis. Although short stems were more frequently implanted in valgus alignment, coronal malalignment itself was not associated with bone resorption in this implant system. Thus, the findings suggest that the implant-specific load-transfer behavior of the evaluated stem design may be more relevant to bone resorption than alignment alone.

Several series have reported localized remodeling predominantly in the greater tuberosity/LM after cementless rTSA.^8^^,^^15^^,^^26^ Other studies have noted greater LD involvement, particularly in association with longer or more diaphyseal-engaging stems.^8^^,^^15^^,^^23^ Additionally, changes in the MM have been described as part of stress shielding adaptations in shoulder arthroplasty cohorts.^8^^,^^21^^,^^22^^,^^32^ The heterogeneous resorption patterns reported across recent rTSA studies may reflect differences in stem geometry, coating distribution, fixation philosophy, and load-transfer characteristics unique to each implant system. Implants with stronger diaphyseal engagement may preferentially unload the LD, whereas metaphyseal-anchoring systems may preserve distal loading while concentrating stress shielding proximally. In our series, the standard stem group exhibited greater LD involvement, which is consistent with the concept that longer stems with greater diaphyseal engagement can divert load away from the proximal and lateral humeral cortex.^7^^,^^17^^,^^18^^,^^24^ In contrast, although LM resorption was also frequent in the short stem group, bone resorption occurred less often in other zones, including the LD and MM zones. Previous studies have shown that metaphyseal-anchoring short stems preserve proximal loading and limit diaphyseal occupancy, thereby reducing distal lateral stress shielding.^7^^,^^17^, ^18^, ^19^^,^^25^ These findings support the concept that stress shielding after rTSA is not a uniform phenomenon but rather an implant-specific remodeling response.

In our study, valgus malalignment occurred in approximately one-third of cases with short stems; however, it was uncommon with standard stems. This finding is consistent with recent comparative data indicating that short metaphyseal-fitting stems have a higher rate of coronal alignment outliers than medium or standard stems used for rTSA.^8^^,^^9^^,^^14^^,^^28^ Short stems provide less diaphyseal self-centering guidance during broaching and impaction; therefore, they may be more sensitive to canal morphology and surgical technique in terms of alignment.^20^ In this context, the geometry of the Equinoxe short stem may have influenced valgus positioning. During insertion, the medial aspect of the proximal stem may contact the medial cortical wall, creating a hinge effect that tends to tilt the stem into valgus. However, coronal malalignment was not independently associated with bone resorption in the present cohort. This observation contrasts with studies involving curved or more diaphyseal-filling stems, in which malalignment and high diaphyseal FR may increase cortical contact and divert load away from the proximal cortex.^14^^,^^27^ The relatively limited distal canal occupation and tapered distal profile of the short stem evaluated in the present study may have reduced the influence of coronal malalignment on proximal load transfer and bone remodeling.

Female sex was associated with bone resorption in this study, consistent with previous reports suggesting that women may be at higher risk of stress shielding-related bone loss because of lower bone mineral density and thinner cortices.^15^^,^^30^^,^^32^ Because bone mineral density was not available in the present database, sex may have served as an imperfect surrogate for skeletal quality. Standard stem use was also associated with bone resorption. Compared with the short stem, the standard stem is longer and has greater diaphyseal engagement, which may concentrate load transfer distally rather than in the proximal metaphysis. Interestingly, canal FRs were not independently associated with bone resorption, in contrast to several previous reports in which higher FR was a strong predictor of stress shielding. One possible explanation is that both stem types in our study demonstrated relatively moderate FRs without excessive distal canal occupation, potentially reducing the influence of FR itself on proximal load transfer. Another explanation is that the use of a single onlay implant platform reduced variability in stem geometry, coating distribution, and fixation concept, thereby attenuating the apparent effect of FR observed in mixed-platform studies. Finally, the higher metaphyseal FR observed in the short stem group may reflect the specific metaphyseal-engaging design of this implant rather than excessive distal fixation, which could explain why higher FR_met did not translate into more extensive resorption. Clinically, we did not detect differences in ROM between shoulders with and without grade ≥3 resorption at 2 years post-operatively. Similar findings have been reported for cementless onlay and standard stems, for which humeral stress shielding and metaphyseal thinning were common but showed little or no association with short- to midterm clinical outcomes.^8^^,^^13^ A recent systematic review also concluded that although stress shielding is prevalent after cementless rTSA, clear clinical consequences have not been consistently demonstrated during short-term follow-up.^30^ The present findings add to the literature by showing that, within a Japanese cohort treated with a single onlay platform, bone resorption was radiographically more frequent with standard stems but was not associated with early ROM differences. Longer follow-up is required to determine whether these radiographic changes influence pain, patient-reported outcomes, periprosthetic fracture risk, fixation durability, or revision complexity.

Our study had some limitations. First, this was a retrospective analysis with a 2-year follow-up period that focused on early remodeling. The long-term clinical significance of grade ≥3 resorption, including its relationship with fixation durability, periprosthetic fracture, and revision complexity, has not yet been determined. Second, propensity score matching can only account for measured covariates and cannot eliminate residual confounding from unmeasured variables, including bone quality, medication use, detailed surgical technique, glenoid-side lateralization, soft tissue tensioning, and post-operative loading conditions. In particular, quantitative data regarding baseplate/glenosphere lateralization were not consistently available, which may have influenced soft-tissue tensioning, humeral load transfer, and bone remodeling. Therefore, the present findings should be interpreted as associative rather than causal. Third, the relatively small sample size after propensity matching may have reduced statistical power, particularly for detecting weaker associations involving alignment and canal filling parameters, and significant findings may be statistically fragile. Fourth, the radiographic evaluation relied on standardized anteroposterior views; therefore, three-dimensional assessments are required. Fifth, bone mineral density measurements were not collected, and sex was likely used as a surrogate for skeletal quality. Sixth, this study was performed in 1 country and included only a single onlay platform; therefore, our results may not be generalizable to inlay geometries, cemented fixation, different stem geometries, other coating distributions, or markedly different neck-shaft angles. Finally, we evaluated only ROM; therefore, pain, patient-reported outcomes, and implant survivorship should be evaluated in future studies.

## Conclusion

In this Japanese cohort treated with a single onlay humeral platform, grade ≥3 humeral bone resorption at 2 years post-operatively was observed more frequently with standard stems than with short stems, particularly in the LD zone. Although short stems were more frequently implanted in valgus alignment, coronal malalignment did not independently predict resorption after adjustment. Female sex and standard stem use were independently associated with bone resorption. These findings appear to be specific to the evaluated prosthesis type and should not necessarily be generalized to other implant types, stem geometries, or fixation concepts.

## Disclaimers:

Funding: No funding was disclosed by the authors.

Conflicts of interest: The author, their immediate family, and any research foundation with which they are affiliated have not received any financial payments or other benefits from any commercial entity related to the subject of this article.
